# Supplementary material for: Electric-Field Mapping of Optically Perturbed CdTe Radiation Detectors
Source: Sensors (Basel). 2023 May 16;23(10):4795. doi: 10.3390/s23104795 (PMC10221137; doi:10.3390/s23104795)
Supplement: Supplementary file 1 [file sensors-23-04795-s001.zip › sensors-2373973-supplementary.pdf]

SUPPLEMENTARY MATERIALS

Electric Field Mapping of Optically Perturbed CdTe Radiation Detectors

Adriano Cola[1*], Lorenzo Dominici[2], Antonio Valletta[3]

[1] Institute for Microelectronics and Microsystems, IMM-CNR, Via Monteroni, 73100, Lecce, Italy
[2] Institute of Nanotechnology, NANOTEC-CNR, Via Monteroni, 73100, Lecce, Italy
[3] Institute for Microelectronics and Microsystems, IMM-CNR, Via Del Fosso Del Cavaliere, 100, 00133, Rome, Italy
* corresponding author: adriano.cola@cnr.it

1. Simulated Electric Field maps/profiles at different instants

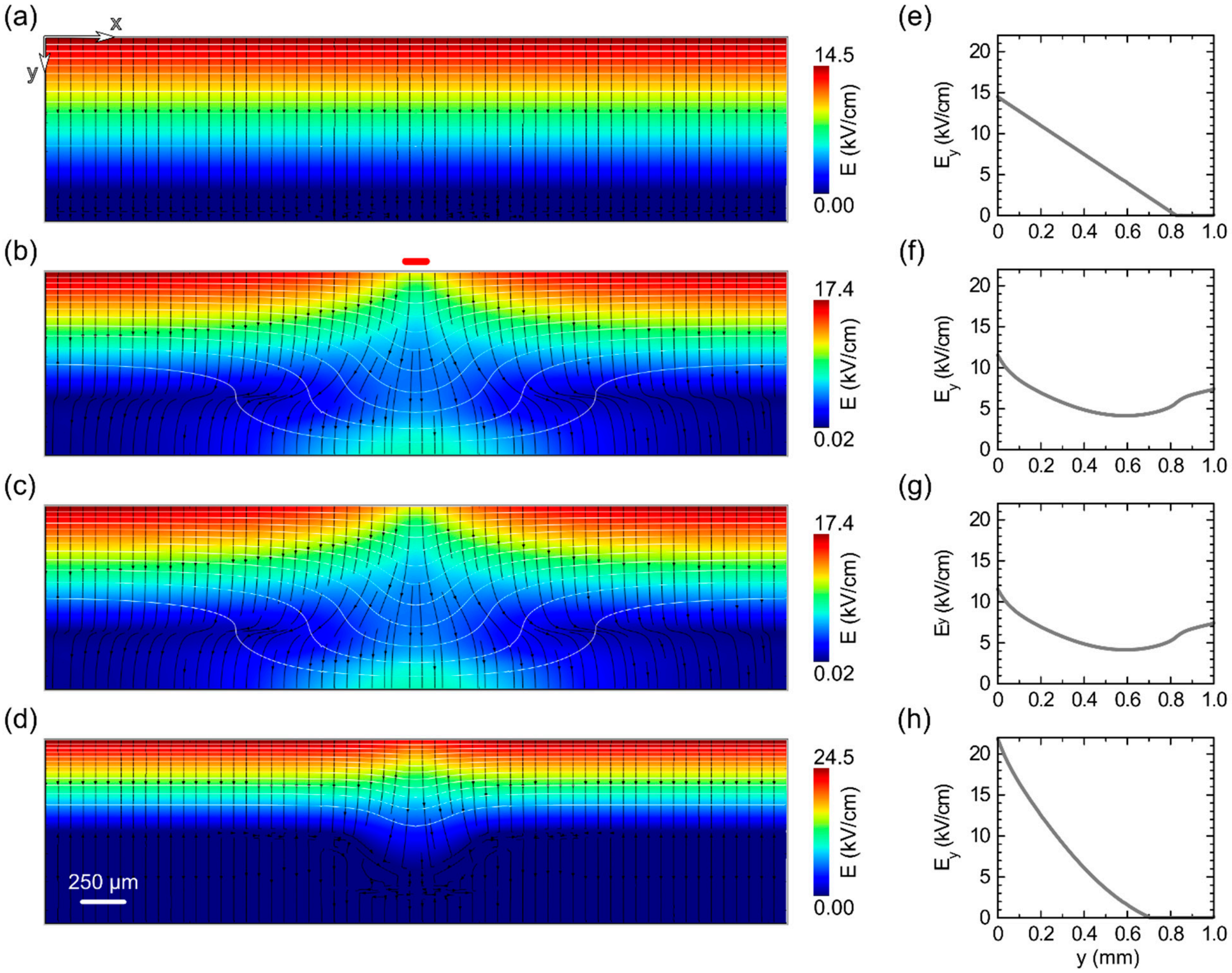

Figure S1 Simulated Electric Field maps corresponding to the experimental ones in Fig.1: a) under dark, just before optical irradiation; b) under light, after 5 min of optical irradiation; c) under dark, 2 sec after the light switch-off; d) under dark 15min after the light switch-off. The corresponding profiles at x=0 are also reported aside each map.

2. Simulated space charge profiles at different instants

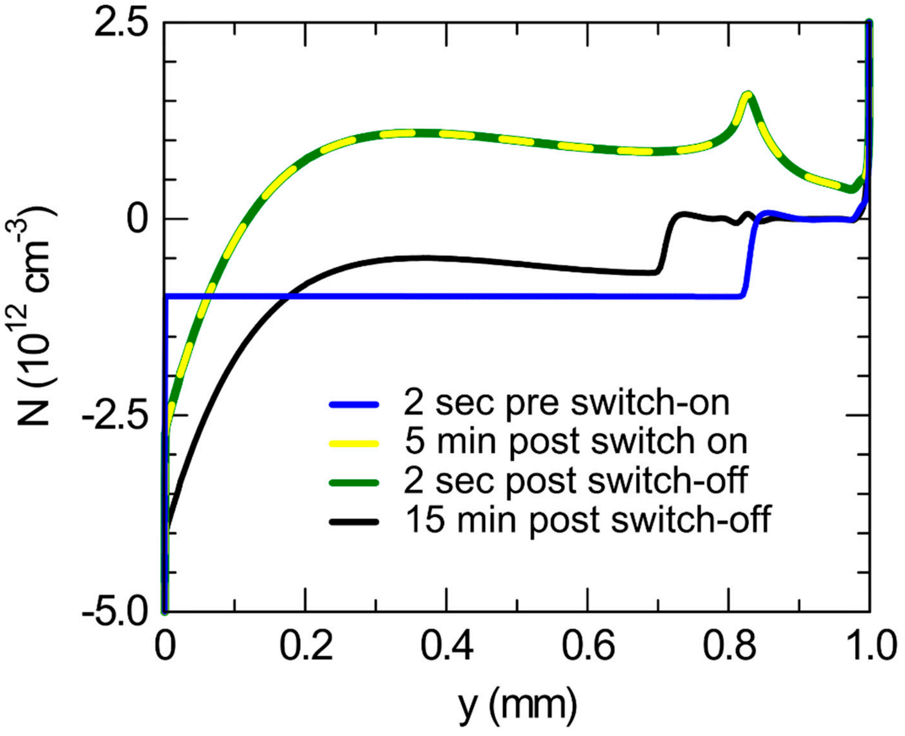

Figure S2 Simulated space charge profiles at x=0 (center of optical irradiation) at different instants: under dark just before light (black), under light, after 5 min of optical irradiation (green), under dark, 15 min after the light switch-off (blue). The peak of positive space charge under light corresponds to the maximum trapping occurring where the electric field becomes negligible near the cathode.

3. Simulation parameters

Table S1. Some relevant parameters of the simulations including those of the 2-level model.

**CdTe**		
Shallow donor level (fully ionized)	Concentration N_d	$2.8x10^{13}cm^{-3}$
Acceptor deep level	Energy E_c-E_a	0.725eV
	Concentration N_a	$5.8x10^{13}cm^{-3}$
	Electr. capture cross section σ_{ea}	$1x10^{-18}cm^2$
	Hole capture cross section σ_{ha}	$1.5x10^{-19}cm^2$
Electron mobility		1000 $cm^2/(Vs)$
Hole mobility		80 cm^2 /(Vs)
Absorption coefficient		Ref. [30]
**In/CdTe/Pt Schottky diode**		
In/CdTe	electron barrier Φ_{In}	0.5eV
Pt/CdTe	electron barrier Φ_{Pt}	0.8eV
**Other parameters**		
Temperature		40°C
